# Genome Sequence of a Mesophilic Hydrogenotrophic Methanogen *Methanocella paludicola*, the First Cultivated Representative of the Order *Methanocellales*

**DOI:** 10.1371/journal.pone.0022898

**Published:** 2011-07-29

**Authors:** Sanae Sakai, Yoshihiro Takaki, Shigeru Shimamura, Mitsuo Sekine, Takahisa Tajima, Hiroki Kosugi, Natsuko Ichikawa, Eiji Tasumi, Aiko T. Hiraki, Ai Shimizu, Yumiko Kato, Rika Nishiko, Koji Mori, Nobuyuki Fujita, Hiroyuki Imachi, Ken Takai

**Affiliations:** 1 Institute of Biogeosciences, Japan Agency for Marine-Earth Science and Technology (JAMSTEC), Yokosuka, Kanagawa, Japan; 2 Department of Environmental Systems Engineering, Nagaoka University of Technology, Nagaoka, Niigata, Japan; 3 Biological Resource Center, National Institute of Technology and Evaluation (NITE), Shibuya-ku, Tokyo, Japan; Max-Planck-Institute for Terrestrial Microbiology, Germany

## Abstract

We report complete genome sequence of a mesophilic hydrogenotrophic methanogen *Methanocella paludicola*, the first cultured representative of the order *Methanocellales* once recognized as an uncultured key archaeal group for methane emission in rice fields. The genome sequence of *M. paludicola* consists of a single circular chromosome of 2,957,635 bp containing 3004 protein-coding sequences (CDS). Genes for most of the functions known in the methanogenic archaea were identified, e.g. a full complement of hydrogenases and methanogenesis enzymes. The mixotrophic growth of *M. paludicola* was clarified by the genomic characterization and re-examined by the subsequent growth experiments. Comparative genome analysis with the previously reported genome sequence of RC-I_MRE50_, which was metagenomically reconstructed, demonstrated that about 70% of *M. paludicola* CDSs were genetically related with RC-I_MRE50_ CDSs. These CDSs included the genes involved in hydrogenotrophic methane production, incomplete TCA cycle, assimilatory sulfate reduction and so on. However, the genetic components for the carbon and nitrogen fixation and antioxidant system were different between the two *Methanocellales* genomes. The difference is likely associated with the physiological variability between *M. paludicola* and RC-I_MRE50_, further suggesting the genomic and physiological diversity of the *Methanocellales* methanogens. Comparative genome analysis among the previously determined methanogen genomes points to the genome-wide relatedness of the *Methanocellales* methanogens to the orders *Methanosarcinales* and *Methanomicrobiales* methanogens in terms of the genetic repertoire. Meanwhile, the unique evolutionary history of the *Methanocellales* methanogens is also traced in an aspect by the comparative genome analysis among the methanogens.

## Introduction

*Methanocella paludicola* is a mesophilic hydrogenotrophic methanogen within the order *Methanocellales*, isolated from rice field soil in Japan [1]. The order *Methanocellales* is the recently proposed euryarchaeotal order, and is composed only of one genus *Methanocella*. Before the taxonomic description of *M. paludicola* and proposal of the order *Methanocellales*, this euryarchaeotal lineage had long been recognized as uncultured archaeal group Rice Cluster I (RC-I). It was indicated by many molecular ecological investigations that the members of RC-I would play a key role in the methane production in rice fields [2].

Based on the phylogenetic analysis using 16S rRNA gene sequences, the methanogens are classified into six orders, *Methanopyrales*, *Methanococcales*, *Methanobacteriales*, *Methanomicrobiales*, *Methanosarcinales* and *Methanocellales* [3], [4]. It is widely accepted that the phylogenetic organization of methanogens generally responds to the taxonomic classification based on morphological, physiological and metabolic traits of a diversity of methanogens [5], [6]. The order *Methanocellales* is closely related to the orders *Methanosarcinales* and *Methanomicrobiales*. The phylogenetic analyses of 16S rRNA gene and the methanogen-specific marker methyl-CoM reductase gene (*mcrA*) sequences suggest that the order *Methanocellales* is the most closely related to the order *Methanosarcinales* [4]. Whereas, the methanogenic substrate utilization of the order *Methanocellales*, that is one of the most important physiological characteristics for methanogens, is similar with the order *Methanomicrobiales* (hydrogenotrophic) rather than the order *Methanosarcinales* (hydrogenotrophic, aceticlastic and methylotrophic). These results suggest that the order *Methanocellales* represents an evolutionary intermediate between the hydrogenotrophic (*Methanomicrobiales*) and versatile [hydrogenotrophic, aceticlastic and methylotrophic] (*Methanosarcinales*) methanogens.

To date, the genome sequences are determined for all the methanogens' orders other than the order *Methanocellales* [7], [8], [9], [10], [11]. The comparative genome investigations have demonstrated new perspectives in evolution of methane-production metabolism and methanogens. For instance, based on the phylogenetic analysis using seven core enzymes of methanogenesis and a protein clustering method using a spectral clustering procedure, Anderson et al. [7] suggested that the methanogenic archaea should be classified into three distinct classes. The Class I consisted of the orders *Methanopyrales*, *Methanococcales* and *Methanobacteriales*, and the Class II and Class III were composed of the order *Methanomicrobiales* and the order *Methanosarcinales*, respectively. Although the genome sequence of RC-I (an environmental genotype of RC-I_MRE50_) is already available by metagenomic approach [12], the coordination between the genomic information and the morphological, physiological and metabolic features characterized in a pure culture is inevitable for the comparative genomic analysis. In this study, therefore, we report the complete genome sequence of *M. paludicola* representing the first pure culture of the order *Methanocellales*. The genome sequence and genomic components are characterized by through the comparison with the previously reported genome sequence of a member of the order *Methanocellales* RC-I_MRE50_ and other methanogens' genomes. The genome sequence of *M. paludicola* would provide new insights into understanding how the members of the order *Methanocellales* (i.e. *M. paludicola* and RC-I_MRE50_) are distinct from each other, and how the order *Methanocellales* is different from other methanogen orders.

## Results and Discussion

### General genome features of *M. paludicola*

**Figure 1:**
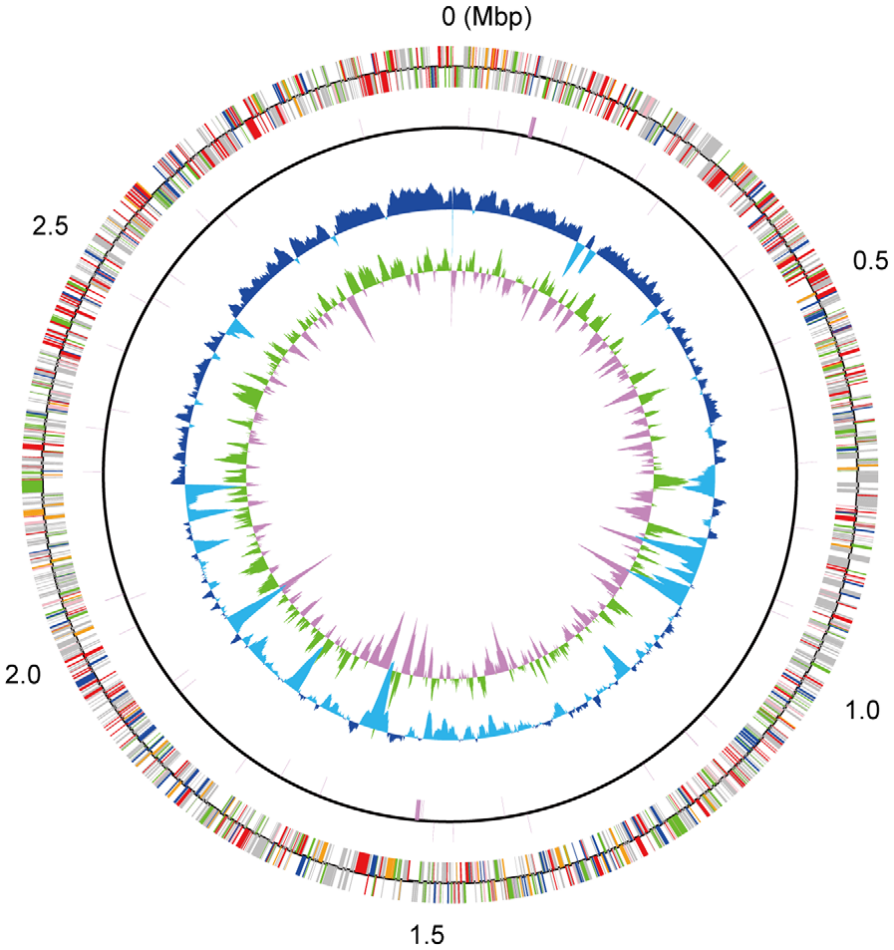
Circular representation of the *M. paludicola* genome. From the inside, the 1st and 2nd circles show the GC skew (values greater than zero are indicated in green and smaller in pink) and the G+C % content (values greater than the average percentage in the overall chromosome or plasmid are shown in blue and smaller in sky blue) in a 10-kb window with 100-b step, respectively. The 3rd and 4th circles show the presence of RNAs (rRNA, tRNA, and small RNA genes) and CDSs aligned in the clockwise and counterclockwise directions are indicated in the upper and lower sides of the circle, respectively. Different colors mean different functional categories: red for metabolism; green for genetic information and processing; blue for membrane transport; orange for cellular processes; pink for miscellaneous and mobile elements, gray for poorly characterized function and purple for RNA genes.

The general features of the *M. paludicola* genome and the previously reported complete genome sequence of RC-I_MRE50_ are listed in Table 1. The genome of *M. paludicola* is a single circular chromosome consisting of 2,957,635 bp in length, which is slightly smaller than the genome of RC-I_MRE50_. Two copies of the 16S-23S-5S rRNA operons with 2 more distantly located 5S rRNA genes are present in the genome of *M. paludicola*. A total of 48 tRNA genes containing putative introns are scattered over the genome. The origin and terminus of replication in the genome of *M. paludicola* was predicted based on cdc6 (MCP_0001) and ORB (origin recognition box). A cluster of repeats was identified in the upstream region of the Cdc6 gene and was contained a mini-ORB like sequence that was found in other archaea [13]. Interestingly, the extremely low G+C content regions are present especially in the lower half of the genome (Fig. 1). The analysis using SIGI-HMM suggested a potential acquisition by horizontal gene transfer. Whereas, the upper half region containing the origin of the genome has the higher G+C content value indicating the relatively well conservation of the upper half of the genome. Indeed, a genome plot analysis of *M. paludicola* and RC-I_MRE50_ revealed a high level of synteny especially near the origin of the genome (see Fig. S1 in the supporting information). The 3004 predicted protein-coding sequences (CDSs) of the *M. paludicola* genome were identified by a combination of coding potential prediction and similarity searches, with an average length of 856 bp, covering 87.4% of the genome. Through the similarity and domain searches of the predicted protein products, specific functions were assigned for 1467 genes (48.8% of the protein coding genes). The remaining 1537 genes (51.2%) were assigned to hypothetical proteins. The distribution of genes into COGs functional categories was not much different between *M. paludicola* and RC-I_MRE50_, however the genes involving in chemotaxis are markedly disparate (Table S1 in the supporting information). The genes for chemotaxis and flagella formation were identified in the genome of RC-I_MRE50_, while it was not found in *M. paludicola*. This finding is in agreement with the characteristic that cell motility was not observed in *M. paludicola*.

**Table 1:** General features of *M. paludicola* and RC-I_MRE50_ genome.

	*M. paludicola*	RC-I _MRE50_
Size (bp)	2,957,635	3,179,916
G+C (%)	54.9	54.6
Protein coding sequences	3004	3085
Contig (%)	87.4	84.7
Function assigned	1467	1604
Function without assigned	1537	1499
rRNA operons	2+5S*2	3
tRNAs (with introns)	48 (4)	54 (5)†
Gene Bank accession no.	AP011532	AM114193

† Three pseudo-tRNAs are included.

### Methanogenesis

**Figure 2:**
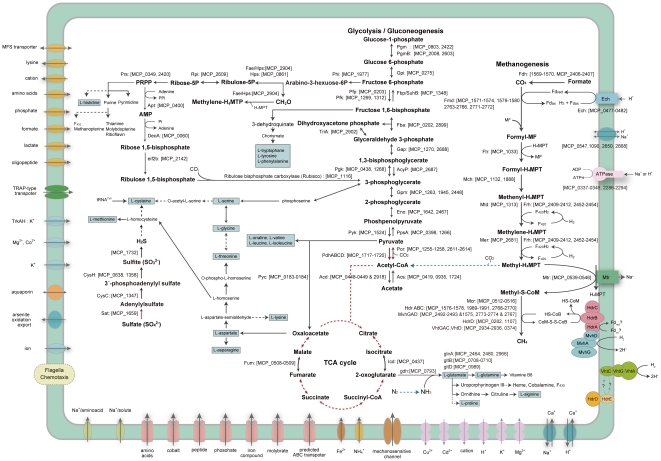
Predicted functional systems and general metabolic pathway of *M. paludicola*. Overview of the presence of genes for homologues of key enzymes of the carbon, sulfur and nitrogen metabolism as well as of selected electron-accepting complexes and transporters. The KEGG database was used for the reconstruction of metabolic pathways. Arrows and dot-lined arrows indicate presence and absence of the genes, respectively. The lines indicate general pathways found in methanogens (black), the unique pathways identified in *M. paludicola* and RC-I_MRE50_ (red), the pathways found in RC-I_MRE50_ but not in *M. paludicola* (blue). Fd, ferredoxin; MF, methanofuran; H_4_MPT, tetrahydromethanopterin; Fdh, formate dehydrogenase; Fmd, formylmethanofuran dehydrogenase; Ftr, formylmethanofuran:H_4_MPT formyltransferase; Mch, methenyl-H_4_MPT cyclohydrolase; Mtd, F_420_-dependent methylene-H_4_MPT dehydrogenase; Mer, methylene-H_4_MPT reductase; Mtr, methyl-H_4_MPT: coenzyme M methyltransferase; Mcr, methyl-coenzyme M reductase; Hdr, heterodisulfide reductase; Ech, energy-converting hydrogenase; Frh, F_420_-reducing hydrogenase; Mvh, non F_420_-reducing hydrogenase; Pgm, phosphoglucomutase; PgmB, ß-phosphoglucomutase; Gpi, glucose-6-phosphate isomerase; SuhB, inositol-1-monophosphatase/fructose-1,6- bisphosphatase; Pfp, pyrophosphate–fructose 6-phosphate 1-phosphotransferase; Pfk, 6-Phosphofructokinase; Fba, fructose-bisphosphate aldolase; TriA, triosephosphate isomerase; Gap, glyceraldehyde-3-phosphate dehydrogenase; AcyP, acylphosphatase; Pgk, phosphoglycerate kinase; Gpm, phosphoglycerate mutase; Eno, enolase; PpsA, phosphoenolpyrivate synthase; Pyk, pyruvate kinase; Por; pyruvate ferredoxin oxidoreductase; Pdh, pyruvate dehydrogenase; Acd, acetyl-CoA synthetase (ADP-forming); Acs, acetyl-CoA synthetase; Pyc, pyruvate carboxylase; Icd, isocitrate dehydrogenase; Fum, fumarate hydratase; Phi, 3-hexulose-6-phosphate isomerase; Fae, formaldehyde-activating enzyme; Hps, 3-hexulose-6-phosphate synthase; Rpi, ribose-5-phosphate isomerase; Prs, ribose-phosphate pyrophosphokinase; Apt, adenine phosphoribosyltransferase; Deo, thymidine phosphorylase; eif2B, translation initiation factor; Sat, sulfate adenylyltransferase; CysC, adenylylsulfate kinase; CysH, phosphoadenosine phosphosulfate reductase.

The *M. paludicola* genome contains sufficient genes to encode a full methanogenesis pathway using H_2_ and CO_2_ (Fig. 2). Similarly to other obligate hydrogenotrophic methanogens, formate dehydrogenase complexes (MCP_1569 to 1570 and MCP_2406 to 2407) and a formate transporter (MCP_2408), a key enzyme for the growth on formate as an alternative methanogenic substrate, were also found in the *M. paludicola* genome. No homologous genes for alcohol dehydrogenase, which is involved in methanogenesis from primary or secondary alcohols, were found. Although an incomplete pathway of potential methanogenesis from methanol was found in the genome of RC-I_MRE50_ [12], none of the corresponding genes for utilizing methanol and other C1 compound was found in the *M. paludicola* genome. The gene structure for the methanogenesis pathway in the *M. paludicola* genome is consistent with the substrate utilization of *M. paludicola* only using H_2_ and CO_2_ or formate as the sole energy source for methane production [4].

Most methanogens contain three distinct types of [NiFe]-hydrogenases, called energy converting hydrogenase, F_420_-reducing hydrogenase and F_420_-nonreducing hydrogenase. Some hydrogenotrophic methanogens additionally contain a [Fe]-hydrogenase, which is also known as H_2_-forming methylene-tetrahydromethanopterin (CH_2_-H_4_MPT) dehydrogenase (Hmd) [14]. All these hydrogenases are involved in methanogenesis pathway [15]. Genes for the hydrogenases except the Hmd were detected in the genome of *M. paludicola*. Among all those hydrogenases, the F_420_-nonreducing hydrogenase co-operates with a heterodisulfide reductase, and reduces heterodisulfide of coenzyme M and coenzyme B (CoM-S-S-CoB) that is synthesized in the last step of methanogenesis pathway. The heterodisulfide reductase/hydrogenase systems are different in between the obligate hydrogenotrophic methanogens such as *Methanothermobacter thermautotrophicus* and the members of the order *Methanosarcinales* [16]. The cytoplasmic cytochrome-lacking F_420_-nonreducing hydrogenase (Mvh) is tightly bound to HdrABC type heterodisulfide reductase. This system is installed in the obligate hydrogenotrophic methanogens [17]. The membrane-bound cytochrome-containing F_420_-nonreducing hydrogenase (Vht) co-operates with the HdrDE type heterodisulfide reductase, which is operative in the member of the order *Methanosarcinales* [18]. Most of the methanogens except for some *Methanomicrobiales* members, which include some members do not possess F_420_-nonreducing hydrogenase, have either of the heterodisulfide reductase/hydrogenase systems [7], [18], [19]. However, both F_420_-nonreducing hydrogenase genes (MCP_2492 to 2493, 1575 and MCP_2773 to 2774, 2767 for Mvh; MCP_2934 to 2936 and 0374 for Vht) were found in the *M. paludicola* genome as well as in the RC-I_MRE50_. The result implies that existence of isozyme systems for heterodisulfide reductase/hydrogenase in the *M. paludicola* and the *Methanocellales* members are atypical of the previously known methanogens.

Since *M. paludicola* is an obligate hydrogenotrophic methanogen and three HdrABC genes for heterodisulfide reductases were found in the genome (MCP_1576 to 1578, MCP_1989 to 1991 and MCP_2768 to 2770), a complete set of the Mvh and HdrABC genes in the *M. paludicola* genome would be associated with the potential *in vivo* function of the heterodisulfide reductase/hydrogenase system. Meanwhile, the membrane-bound cytochrome-containing F_420_-nonreducing hydrogenase (Vht) co-operates with the HdrDE type heterodisulfide reductase. The homolog gene for HdrD, which contained the catalytic site for the reduction of the disulfide substrate [20], was found in the *M. paludicola* genome (MCP_0282). This gene conserved two CCG domains and two [4Fe-4S] clusters as observed in the HdrD gene of *Methanosarcina barkeri* [20], [21]. Thus, it seems likely that the possible *M. paludicola* HdrD would have the same function as the *Methanosarcinales* HdrD. However, none of the homolog genes for the potential HdrE that contained a b-type cytochrome and interacted with electron donor was present in the *M. paludicola* genome. The absence of HdrE is common between the *Methanocellales* genomes of *M. paludicola* and RC-I_MRE50_. As the active site for heterodisulfide reductase activity is conserved in the HdrD, the heterodisulfide reductase/hydrogenase system (Vht/HdrD) may be operative in *M. paludicola* without the potential HdrE function. In *Methanosarcina* members, the heterodisulfide reductase/hydrogenase system (Vht/HdrDE) builds up an electrochemical gradient, then proton translocating ATP synthetase synthesizes ATP from the generated proton motive force [22]. Phylogenetic analysis of the ATP synthetases in *M. paludicola* (MCP_0339) and RC-I_MRE50_ (RCIX2030) indicated that ATP synthetases of *Methanocellales* members more closely related to the ATP synthase of *Methanosarcina mazei* (MM_0780) (see Fig. S2 in the supporting information), which was experimentally confirmed as proton-translocating ATP synthase [23]. Moreover, the alignment of ATP synthase showed that the gene of *M. paludicola* does not contain sodium-binding site that was characterized in *Ilyobacter tartaricus* [24] (see Table S2 in the supporting information). This sodium-binding site was not also found in the ATP synthase of *M. mazei*, while it was found in the ATP synthase of *M. thermautotrophicus*, which possess the experimentally characterized sodium-translating ATP synthase [25]. Therefore it seems more likely that *M. paludicola* possess proton-translating ATP synthase. Moreover, Ech hydrogenase, which provides reducing equivalent of the first step of methanogenesis from H_2_ and CO_2_ in *M. barkeri* [26], is present in *M. paludicola* (MCP_0477 to 0482) as well as RC-I_MRE50_. Ech hydrogenase is also assumed to be proton-translocating [22]. Therefore *M. paludicola* and *Methanocellales* members seem likely to use proton-translocating system. The *Methanosarcina*-like heterodisulfide reductase/hydrogenase system (Vht/HdrD) is more likely to operate in *M. paludicola* and *Methanocellales* members. Nevertheless further biochemical studies are necessary to clarify the in vivo function of these genes.

### Central metabolism

The lack of genes encoding carbon monoxide dehydrogenase/acetyl-CoA synthase (CODH/ACS) is a distinctive feature of the *M. paludicola* genome (Fig. 2). Those genes are present in RC-I_MRE50_ genome (LRC456–463). Thus the capability of acetate assimilation may be different between the two members of the *Methanocellales* methanogens. Since *M. paludicola* does not possess CODH/ACS, it metabolically loses a bypass between methyl-tetrahydromethanopterin (CH_3_-H_4_MPT) and acetyl-CoA and is not able both to assimilate CO_2_ as the sole carbon source and to utilize acetate as the methanogenic source. It was pointed out as a notable growth characteristic that *M. paludicola* required acetate for the growth [4]. The genome harbors the genes for acetyl-CoA metabolisms, e.g. AMP forming acetyl-CoA synthases (MCP_0419 and MCP_0935) and ADP forming acetyl-CoA synthetases (MCP_0448 to 0449, and MCP_2918). As predicted in *Methanosphaera stadtmanae* [27], acetate is likely assimilated into acetyl-CoA from which many cell compounds could be synthesized. Indeed, tracer experiments using the ^13^C-labeled acetate or bicarbonate showed that both acetate and bicarbonate were incorporated into the growing cells of *M. paludicola*, while acetate was more abundantly assimilated than bicarbonate (see Table S3 for the supporting information). These results indicate that *M. paludicola* require acetate as the primary carbon source but also mixotrophically grows with acetate and CO_2_. The carbon dioxide serves not only as the absolute electron acceptor for methanogenesis (energy source) but also as the additional carbon source. Carbon dioxide might accessorily be incorporated by the following reactions, such as pyruvate ferredoxin oxidoreductase (Por) and ribulose-1,5-bisphosphate carboxylase-oxygenase that involve in pyruvate metabolism and AMP metabolism, respectively (Fig. 2).

It was noteworthy that most of the genetic components for tricarboxylic acid (TCA) cycle were absent in the *M. paludicola* genome (Fig. 2). The oxaloacetate can be generated from pyruvate by pyruvate carboxylase (MCP_0183 and 0184). However, as similar to the RC-I_MRE50_ genome, the only two enzymes, isocitrate dehydrogenase (MCP_0437) and fumarase (MCP_0508 and 0509) seemed to be encoded. Therefore *M. paludicola* is not able to synthesize 2-oxoglutarate, the precursor of glutamate. Generally, in many methanogens, the incomplete TCA cycle starts from oxaloacetate to proceeds in either reductive or oxidative direction leading to 2-oxoglutarate. It is suggested that the members of the order *Methanosarcinales* adopt the oxidative direction of the TCA cycle mediated by the enzymes of citrate synthase, aconitase, and isocitrate dehydrogenase [28]. In contrast, the reductive pathway of TCA cycle would operate in the obligately hydrogenotrophic methanogens for the production of 2-oxoglutarate via malate, fumarate, and succinate [29]. However, as described above, neither pathway was reconstructed from the *M. paludicola* genome. The reconstructed TCA cycle from the genome sequence let us to predict that *M. paludicola* may require some intermediates of TCA cycle or their derivatives such as 2-oxoglutarate and L-glutamate. Although the previous study described that yeast extract was not absolutely required for the growth of *M. paludicola* [4], it has recently become evident that the complete lack of supplementation of yeast extract finally results in the discontinuity of *M. paludicola* culture. The further growth experiments indicated that the supplementation of yeast extract was compensated with L-glutamate. Therefore, *M. paludicola* requires L-glutamate for their growth. The CDSs for amino acid transporter (MCP_2742 to 2744) were found in the genome. Due to their quite incomplete genetic sets of TCA cycle, *M. paludicola* and its relatives probably require at least L-glutamate for the growth as a donor of amino groups.

The absence of the TCA cycle in *M. paludicola* also indicates the inability to produce NADH in addition to amino acids. However, *M. paludicola* encodes pyruvate dehydrogenase (MCP_1717 to 1720), which catalyzes the NAD-linked oxidative decarboxylation of pyruvate concomitantly with the formation of acetyl-CoA (Fig. 2). Although the pyruvate dehydrogenase has usually been found in aerobe and facultative anaerobes, *M. paludicola* as well as RC-I_MRE50_ genomes encoded the enzyme. Since two genomes lack the genes for TCA cycle, pyruvate dehydrogenase might function as the alternative system for NADH production.

### Nitrogen and Sulfur metabolism

Nitrogen fixation is catalyzed by a nitrogenase consisting of two components: dinitrogenase reductase called Fe protein encoded by *nifH* and dinitrogenase called MoFe protein encoded by *nifDK* [30]. Although a full component of the genes for nitrogenase were found in RC-I_MRE50_, only homologues of *nifH* were found in *M. paludicola* (MCP_0364 and MCP_0905). The *nifH* genes are widely found in many methanogens regardless of their nitrogen fixation capability. It has been suggested that the *nifH* gene product of non-N_2_-fixing methanogen is not associated with the N_2_-fixing function but may be involved in the biosynthesis of factor 430 [31]. In fact, the phylogenetic analysis showed that the *nifH* genes of *M. paludicola* clustered with the *nifH* genes from non-N_2_-fixing methanogens (see Fig. S3 in the supporting information). Since *M. paludicola* possess an ammonium transport system (MCP_0585–0588), it would take in ammonium from outside the cell. Meanwhile, one of the *nifH* gene of RC-I_MRE50_ was clustered with the *nifH* genes from nitrogen-fixing bacteria *Clostridium acetobutylicum* [32] (see Fig. S3 in the supporting information), and the *nifH* gene was part of an operon with other *nif* genes e.g. *nifE*, *nifK*. Therefore the nitrogen fixation capability might be an inter-species physiological difference among the member of the order *Methanocellales*.

Although many methanogens are recognized to be sensitive to sulfite [33], some methanogens not only tolerate the existence of sulfite but also utilize sulfite as a sole sulfur source [34], [35]. To date, the reduction of sulfate to sulfide has been reported only for *Methanothermococcus thermolithotrophicus* [34]. Nevertheless, the genetic and enzymatic components remain uncertain. The potential assimilatory sulfate reduction pathway from sulfate to sulfide was reconstructed in the *M. paludicola* genome (Fig. 2). The CDSs for adenylyltransferase (MCP_1659), adenylylsulfate kinase (MCP_1347), phosphoadenosine phosphosulfate reductase (MCP_0638 and MCP_1358) and sulfite reductase (MCP_1732) were identified. Meanwhile, we did not find the genes for a coenzyme F_420_-dependent sulfide reductase that was characterized in *M. jannaschii* [36]. These genetic components for potential assimilatory sulfate reduction pathway shared both in the *M. paludicola* and RC-I_MRE50_ genomes. All other previously reported genomes of methanogens lack the genes encoding adenylyltransferase. Moreover, the adenylylsulfate kinase was encoded only in the genomes of *M. paludicola* and RC-I_MRE50_ and *Methanosaeta thermophila*. Although the sulfur detoxification and assimilation of the *Methanocellales* members should be experimentally characterized by the growth experiments, the genome-predicting sulfur metabolisms represent one of the distinctive features of the *Methanocellales* members.

### Antioxidant systems

Methanogens are usually characterized by the most severe anaerophilia of all the life in this planet. Thus, it has been believed that the ecological niches of methanogens are quite limited in the O_2_-abundant modern earth. However, a few species are known to be relatively resistant to the O_2_ exposure [37], [38]. In such methanogens, possible antioxidant systems such as superoxide dismutase [39], catalase [40] and F_420_H_2_ oxidase [41] have been characterized. In addition, different kinds of antioxidant enzymes have been identified in bacteria and other archaea, e.g. superoxide reductase including neelaredoxin, desulfoferrodoxin and desulforedoxin, and peroxiredoxins including alkyl hydroperoxide and thiol-specific peroxidase [42]. The candidate genes coding these antioxidant enzymes in the *M. paludicola* and other methanogens' genomes are listed (see Table S4 in the supporting information). The *M. paludicola* genome encoded superoxide reductase (MCP_0733) and rubredoxin (MCP_2757), which could catalyze the reduction of superoxide to hydrogen peroxide, and rubrerythrin (MCP_0328 and MCP_2368) and peroxiredoxins (MCP_0070, MCP_0461, MCP_0633 and MCP_2051) for detoxification of hydrogen peroxide to water. In addition, F_420_H_2_ oxidase (FprA) (MCP_2921) would be involved in O_2_ detoxification by catalyzing a four-electron reduction of O_2_ to H_2_O [41]. Although, RC-I_MRE50_ genome additionally encodes the genes for superoxide dismutase, catalase and desulfoferrodoxin, none of the homologous genes were found in *M. paludicola*. The greater genetic complement for antioxidant enzymes of the RC-I_MRE50_ genome may point that the antioxidant system might be different among the *Methanocellales* members. Since superoxide dismutase and catalase are generally found in aerobes and facultative anaerobes, the absence of those genes in the *M. paludicola* genome indicates that *M. paludicola* would more prefer to inhabit strictly anaerobic condition, and needs the apparatus for monitoring oxygen concentration in their habitats. Actually, as similar to other methanogen genomes [43], *M. paludicola* possesses histidine kinase containing PAS domains that can be involved in sensing the redox condition changes [44]. The domain architectures of histidine kinases of *M. paludicola* and RC-I_MRE50_ differ in pattern (see Table S5 in the supporting information). It also suggests that antioxidant system might be different between the two methanogens.

### Comparative genomics

The whole-genome-level features of *M. paludicola* were characterized by comparative analyses with the closely related genotype (RC-I_MRE50_) and with the previously determined methanogens' genomes. The *M. paludicola* genome shared 70% of the CDSs with those of RC-I_MRE50_ genome. When a similar comparison was conducted in the genomes of different species within the genus *Methanosarcina*, the percentage was in the range of 62–73% (Data not shown). Thus, it seems likely that the genetic composition is well correlated with each other within the *Methanocellales* members as well as *Methanosarcina* species. Meanwhile, in the comparison with the genomes from other orders of methanogens, relatively high percentage was obtained from the comparisons with the members of the orders *Methanosarcinales* (37.3–45.6%) and *Methanomicrobiales* (35–42.7%), while the value with other orders was less than 33% (see Table S6 in the supporting information). These results strongly suggested the structural and compositional relatedness of the *Methanocellales* genomes with the *Methanosarcinales* and *Methanomicrobiales* genomes. Although the phylogenetic relatedness of the *Methanocellales* with the *Methanosarcinales* and *Methanomicrobiales* methanogens has been often pointed to by the phylogenetic analyses of rRNA and methyl-CoM reductase genes, the genomic compositional relatedness provides further insight into understanding the evolution of these modern lineages of methanogens.

**Figure 3:**
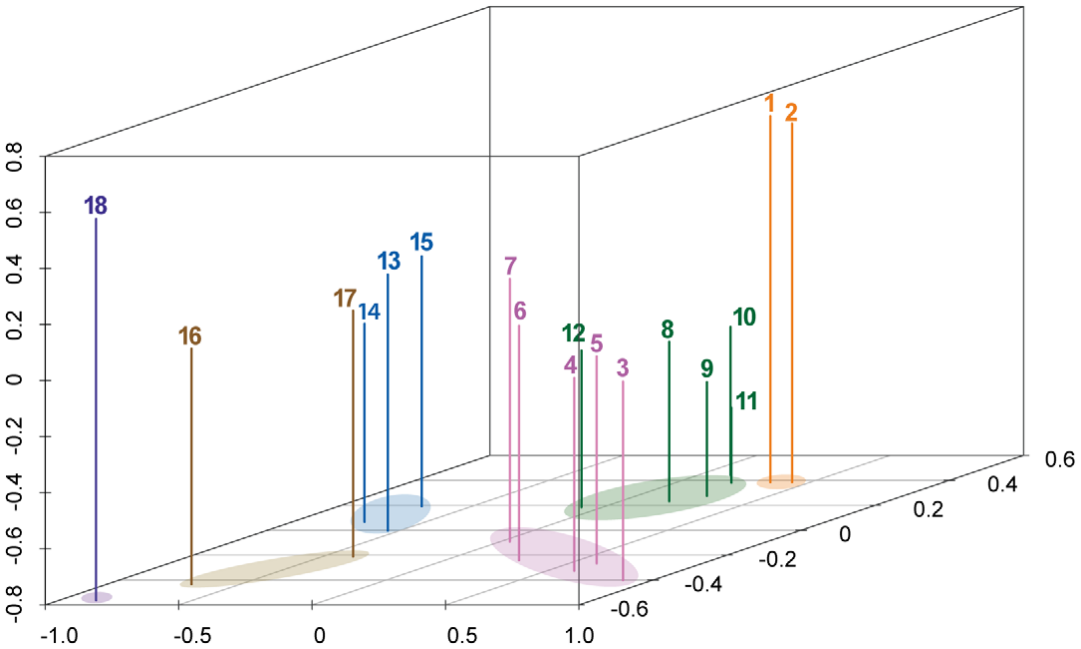
The ordination plot of genomes of methanogenic archaea using non-metric multidimensional scaling. Distances were calculated from gene profiles of 18 representative archaeal genomes, based on protein families by the method described in Materials and Methods. The organisms are color-coded according to their affiliated orders: orange, *Methanocellales*; red, *Methanosarcinales*; green, *Methanomicrobiales*; blue, *Methanobacteriales*; brown, *Methanococcales*; purple, *Methanopyrales*. The organisms are represented with the numbers: 1, *M. paludicola* SANAE; 2, uncultured methanogenic archaeon RC-I, RC-I_MR50_; 3, *M. a acetivorans* C2A; 4, *M. barkeri* Fusaro; 5, *M. mazei* Go1; 6, *M. burtonii* ACE-M; 7, *M. thermophila* PT; 8, *M. marisnigri*; 9, *M. palustris* E1-9c; 10, *M. boonei* 6A8; 11, *M. hungatei* JF-1; 12, *M. labreanum* Z; 13, *M. thermautotrophicus* ΔH; 14, *M. smithii* PS; 15, *M. stadtmanae* MCB-3; 16, *M. jannaschii* JAL-1; 17, *M. maripaludis* S2; 18, *M. kandleri* AV19.

In addition, the ordination of methanogens' genomes was generated using non-metric multidimensional scaling (NMDS) method (Fig. 3), which is a major branch of multivariate analysis. The NMDS ordinations attempt to place all samples in an arbitrary three-dimensional space, in which the relative distances between the samples indicate the corresponding pairwise similarity. Hence, the closely related organisms in the NMDS ordination would have the similar gene repertoires. In the NMDS map, the *Methanocellales* genomes made an independent group from other methanogen genomes, and were most closely related with the *Methanomicrobiales* genomes while the relatively closer relationship between the *Methanocellales* and the *Methanosarcinales* was also represented (Fig. 3). Since the highly conserved genetic elements are potentially relevant to the important physiological functions, the closer relationship in the NMDS map may represent the greater similarity in the conservative cellular functions and metabolisms, and the ecological niches. Thus, the close relationship of the *Methanocellales* genomes with the *Methanomicrobiales* and *Methanosarcinales* genomes in the NMDS map strongly suggests the general physiological relatedness of the *Methanocellales* to both *Methanomicrobiales* and *Methanosarcinales* methanogens. Interestingly, however, the *Methanocellales* genomes represented the unique position with height direction. The distinctiveness of the position is comparable to that of the *Methanopyrus kandleri* genome [10], of which the physiological uniqueness is characterized by the most hyperthermophily [45] and potentially the most ancient lineage of life [46]. This result suggests that the *Methanocellales* methanogens would also have some of the exclusive characteristics from any other methanogenic groups. Insights from these genome-wide relationships and the molecular phylogenetic relationships among the *Methanomicrobiales*, *Methanocellales* and *Methanosarcinales* members will shed light on the evolution of these modern lineages of methanogens.

## Materials and Methods

### *Methanocella paludicola* strain SANAE

The detailed physiological description of *M. paludicola* strain SANAE (NBRC 101707, JCM 13418 and DSMZ 17711) was reported previously [4].

### Genome sequencing, assembly, and gap closure

The genome of *M. paludicola* was sequenced using a conventional whole-genome shotgun strategy following the method of Takarada et al. [47]. All plasmid clones were end-sequenced by using dye-terminator chemistry on an ABI Prism 3730 sequencer. Raw sequence data corresponding to approximately 10-fold coverage was assembled by using PHRED/PHARAP/CONSED software (http://www.pharap.org) [48]. Gaps between the assembled sequences were primarily closed by primer walking on gap-spanning library clones or with PCR products from genomic DNA. Assessment of final assembly quality was completed as described previously [47].

### Gene identification and annotation

Putative non-translated genes were identified by using Rfam [49], tRNAscan-SE [50], ARAGON [51] and SPRITSX [52]. While, rRNA was identified by the BLASTN program [53]. The potential protein sequences were predicted using a combination of GLIMMER [54] and GeneMarkS [55], and start sites were manually inspected and altered. These predicted CDSs were translated and were searched against the UniProt database [56] and the protein signature database, InterPro [57]. The KEGG database [58] was used for pathway reconstruction. Signal peptides in proteins were predicted by using SIGNALP 3.0 [59], and transmembrane helices were predicted by using TMHMM [60]. Horizontal gene transfer was predicted by using SIGI-HMM [61].

### Incorporation of ^13^C-labeled acetate and bicarbonate into cell material

The cultivation for *M. paludicola* followed the procedure previously described [4]. Basal medium contained 1 mM acetate and 30 mM NaHCO_3_. The ^13^C labeled acetate or bicarbonate was added to the culture at a final concentration of 5% (w/w) of non-labeled substrate. All cultivations were performed in the 120-ml serum vials containing 40 ml medium (pH 7.0 at 25°C) under an atmosphere of H_2_/CO_2_ (80/20 [v/v]). The temperature for cultivation was maintained at 37°C. Cells were corrected in the logarithmic growth phase with grass micro filter. The filtered cells were washed with 20 ml of 1.5% (w/v) NaCl solution containing 1 M HCl and were then washed with 20 ml 1.5% (w/v) NaCl solution. The filters were frozen and lyophilized. The incorporation of ^13^C-labeled acetate and bicarbonate in the lyophilized cells were examined by elemental-analysis-isotope-ratio-mass-spectroscopy (EA-IRMS). The stable isotope composition was determined by SI Science (Saitama, Japan) using isotope ratio mass spectrometer DELTA plus Advantage (Thermo Fisher Scientific).

### Glutamate auxotrophy test

To check glutamate auxotrophy of *M. paludicola*, L-glutamate (0.5 mM), instead of yeast extract, was added to the medium. Growth was determined by monitoring the concentration of methane by using a GC-3200G gas chromatograph (GL Science) with a thermal conductivity detector. The measurements were performed in duplicate.

### Comparative genomic analysis

For comparative genome analysis, following methanogenic archaeal genomes were used: *Methanopyrus kandleri* AV19 (AE009439), *Methanocaldococcus jannaschii* JAL-1 (L77117), *Methanococcus maripaludis* S2 (BX950229), *Methanothermobacter thermautotrophicus* ΔH (AE000666), *Methanobrevibacter smithii* PS (CP000678), *Methanosphaera stadtmanae* MCB-3 (CP000102), *Methanocorpusculum labreanum* Z (CP000559), *Methanospirillum hungatei* JF-1 (CP000254), *Methanoculleus marisnigri* JR1 (CP000562), *Methanosphaerula palustris* E1-9c (CP001338), *Methanoregula boonei* 6A8 (CP000780), *Methanosarcina acetivorans* C2A (AE010299), *Methanosarcina barkeri* Fusaro (CP000099), *Methanosarcina mazei* Go1 (AE008384), *Methanococcoides burtonii* ACE-M (CP000300), *Methanosaeta thermophila* PT (CP000477) and uncultured methanogenic archaeon RC-I, RC-I_MRE50_ (AM114193). Proteome for all analyzed methanogens was clustered by the FORCE program [62]. In order to examine genome-wide relationship of *M. paludicola* in term of gene repertories, the ordination of archaeal genomes was generated using non-metric multidimensional scaling (NMDS) method, which is a one of the method of multivariate analysis. NMDS ordinations attempt to place all samples in an arbitrary three-dimensional space, in which the relative distances between the samples indicate the corresponding pairwise similarity. Therefore, the closely related organisms in the NMDS ordination would have the similar gene repertories. Gene context of each genome were constructed as described previously [63].

### Nucleotide sequence accession number

The sequence and annotation of the complete *M. paludicola* genome is available at GeneBank/EMBL/DDBJ under accession numbers AP011532.

## Supporting Information

Figure S1 **Genome plot of the orthologous gene pairs between the genomes of** ***M. paludicola*** **and RC-I_MRE50_.** Pairwise ortholog families were identified with the InParanoid program (Remm et al., 2001, J. Mol. Biol., 314: 1041–1052). Orange circle indicates the area peripheral to the origin of the DNA replication.
(TIF)

Figure S2 **Phylogenetic tree of Na^+^-translocating and proton-translocating A_1_A_0_-ATPases in methanogenic archaea.** The neighbor-joining phylogenetic tree was constructed on the basis of a sequence alignment of A subunits of ATPases. The names of microbes with experimentally characterized ATPases are shown in colored red for Na^+^-dependent enzymes and blue for H^+^-dependent enzymes. The accession numbers are shown in parentheses after each sequence name. The scale bar indicates the estimated number of base changes per amino acid position. The numbers at internal branches indicate the bootstrap probabilities with 1,000 resampled data sets.
(TIF)

Figure S3 **Phylogenetic tree of nifH-deduced amino acid sequences showing the phylogenetic position of** ***M. paludicola*** **(indicated by bold type).** The tree was constructed by the neighbor-joining method using the ARB software package (Ludwig et al., 2004, Nucleic Acids Res., 32: 1363–1371). The accession numbers are shown in parentheses after each sequence name. The scale bar indicates the estimated number of base changes per amino acid position. The symbols at branch nodes indicate bootstrap values. Bootstrap analysis was performed with 1,000 resampled data sets.
(TIF)

Table S1 Numbers of genes associated with the general COG functional categories.
(PDF)

Table S2 Sequence alignment of c/K subunits of V type ATPases. This table was made based on the data of Mulkidjanian *et al.* [24]. Active site residues are indicated in colored as follows: conserved ion-binding acidic (Glu/Asp) residue in red; other Na^+^ ligands are in right blue. The hydrophobic residue corresponding to Val63 of *Ilyobacter tartaricus* c subunit is colored orange. The conserved small (Pro, Gly, Ala, Ser) residue, corresponding to Pro28 of *I. tartaricus* c subunit is colored pink. Predicted cation specificity of the c/K subunit. Ions whose binding has been experimentally studied are shown in bold.
(PDF)

Table S3 Carbon isotope fractionation of *M. paludicola*. Control indicates the value for the non- labeled cells.
(PDF)

Table S4 Antioxidant enzymes among methanogens. The number indicates multiple gene copies. -, not present; n.d., not determined. Catalases indicated are E (katE, type I monofunctional clade II large subunit hemed catalase), A (katA, type I monofunctional clade III small subunit hemeb catalase), and G (katG, type II bifunctional hemeb catalase/peroxidase). Type of superoxide dismutases indicated are C (sodC, Cu-Zn-containing periplasmic enzyme) and B (sodB, Fe-containing cytoplasmic enzyme).
(PDF)

Table S5 The number of genes of histidine kinase and response regulator of *M. paludicola* and RC-I_MRE50_.
(PDF)

Table S6 Distribution of shared genes of *M. paludicola* and RC-I_MRE50_ with other methanogenic archaeal genomes. Asterisks show the value indicates the number and percentage of the genes of *M. paludicola* and RC-I_MRE50_ shared with each methanogen genome.
(PDF)
