# Supplementary figures and images for: Genome Sequence of a Mesophilic Hydrogenotrophic Methanogen Methanocella paludicola, the First Cultivated Representative of the Order Methanocellales
Source: PLoS One. 2011 Jul 29;6(7):e22898. doi: 10.1371/journal.pone.0022898 (PMC3146512; doi:10.1371/journal.pone.0022898)

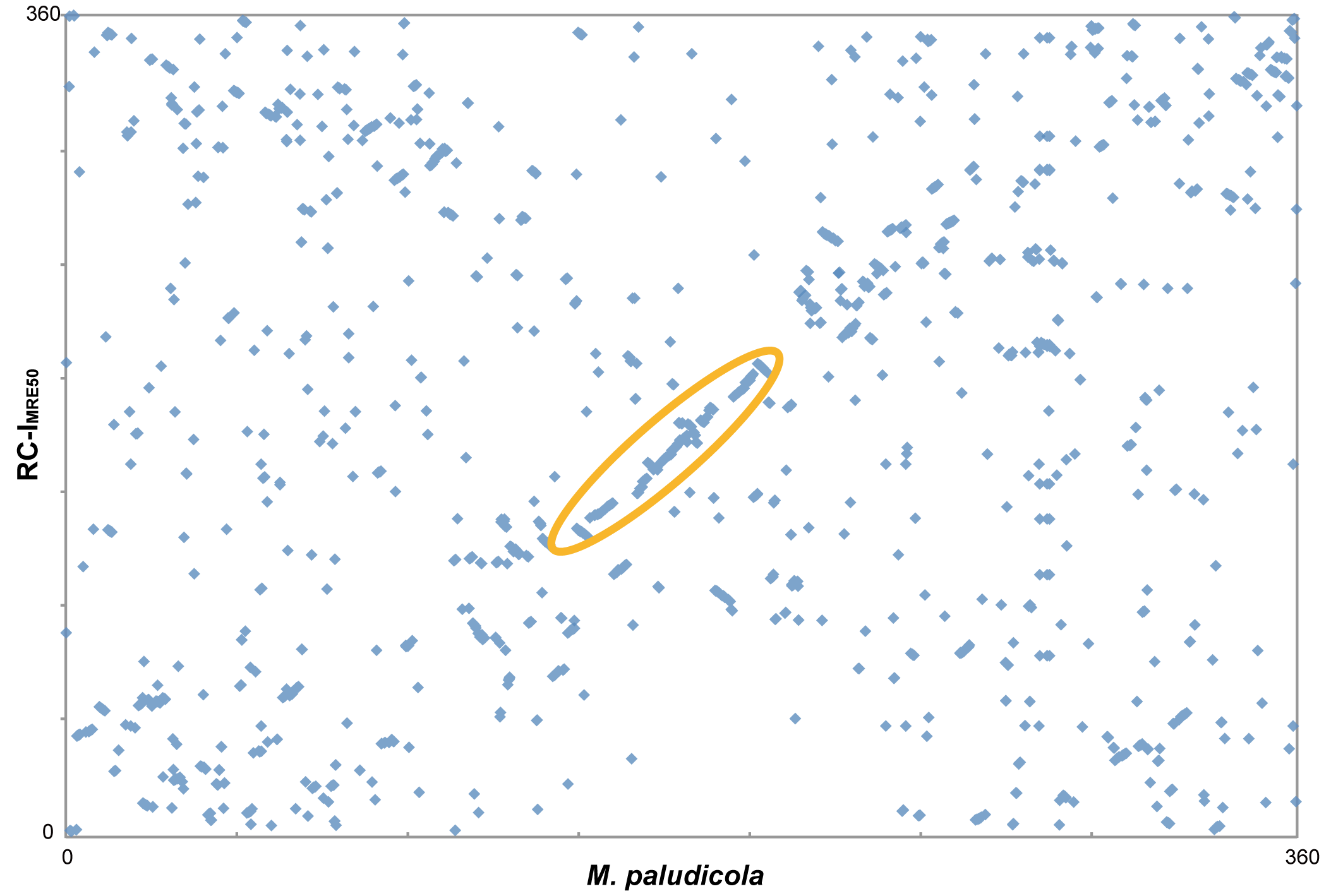

Supplement: Figure S1 — Genome plot of the orthologous gene pairs between the genomes of M. paludicola and RC-IMRE50. Pairwise ortholog families were identified with the InParanoid program (Remm et al., 2001, J. Mol. Biol., 314: 1041–1052). Orange circle indicates the area peripheral to the origin of the DNA replication. (TIF) [file pone.0022898.s001.tif]

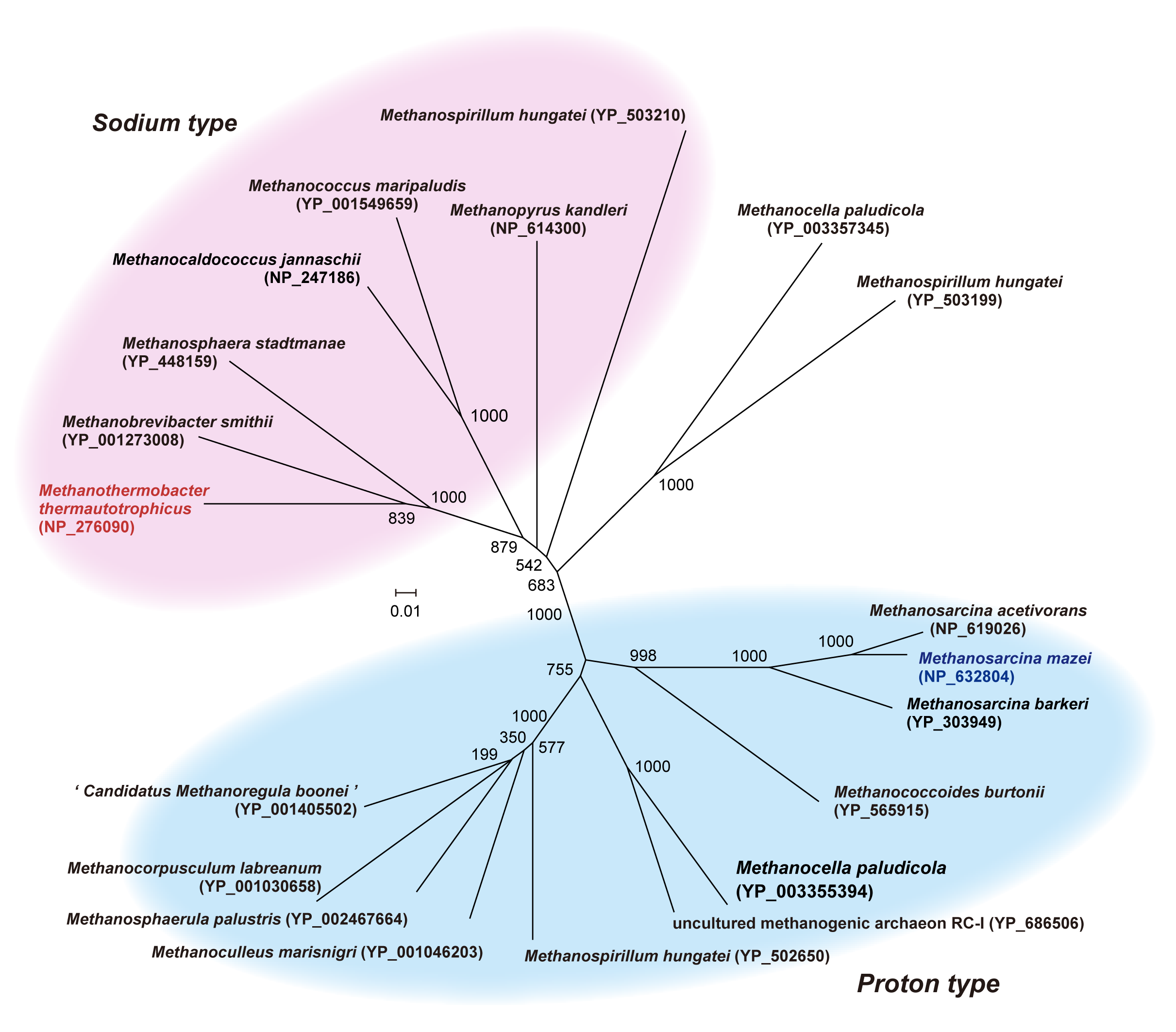

Supplement: Figure S2 — Phylogenetic tree of Na+-translocating and proton-translocating A1A0-ATPases in methanogenic archaea. The neighbor-joining phylogenetic tree was constructed on the basis of a sequence alignment of A subunits of ATPases. The names of microbes with experimentally characterized ATPases are shown in colored red for Na+-dependent enzymes and blue for H+-dependent enzymes. The accession numbers are shown in parentheses after each sequence name. The scale bar indicates the estimated number of base changes per amino acid position. The numbers at internal branches indicate the bootstrap probabilities with 1,000 resampled data sets. (TIF) [file pone.0022898.s002.tif]

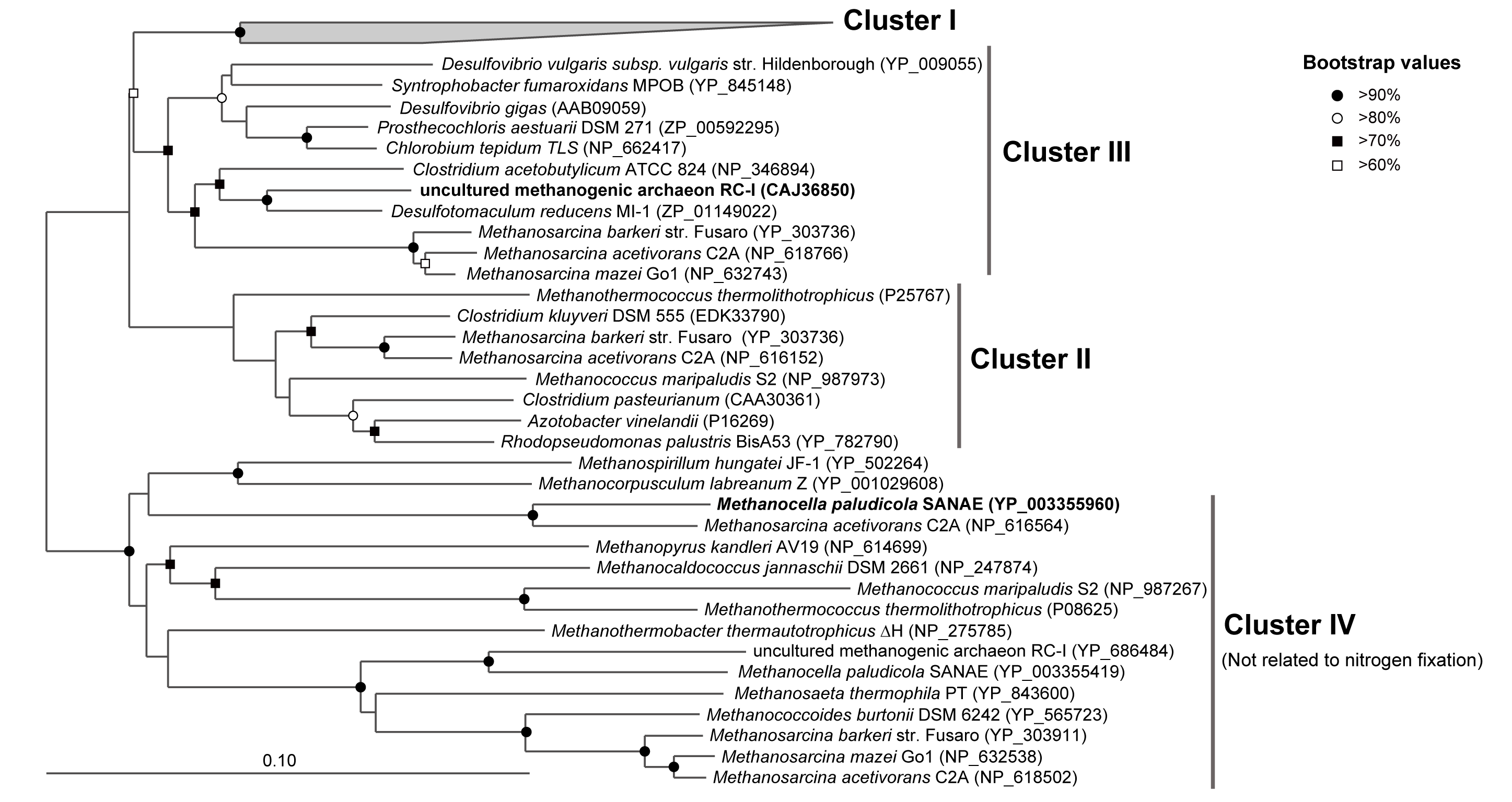

Supplement: Figure S3 — Phylogenetic tree of nifH-deduced amino acid sequences showing the phylogenetic position of M. paludicola (indicated by bold type). The tree was constructed by the neighbor-joining method using the ARB software package (Ludwig et al., 2004, Nucleic Acids Res., 32: 1363–1371). The accession numbers are shown in parentheses after each sequence name. The scale bar indicates the estimated number of base changes per amino acid position. The symbols at branch nodes indicate bootstrap values. Bootstrap analysis was performed with 1,000 resampled data sets. (TIF) [file pone.0022898.s003.tif]
