# Supplementary material for: Genome Sequence of a Mesophilic Hydrogenotrophic Methanogen Methanocella paludicola, the First Cultivated Representative of the Order Methanocellales
Source: PLoS One. 2011 Jul 29;6(7):e22898. doi: 10.1371/journal.pone.0022898 (PMC3146512; doi:10.1371/journal.pone.0022898)
Supplement: Table S1 — Numbers of genes associated with the general COG functional categories. (PDF) [file pone.0022898.s004.pdf]

Table S1. Numbers of genes associated with the general COG functional categories.

Code	Description	Value	
		M. paludicola	RC-I_{MRE50}
J	Translation, ribosomal structure and biogenesis	158	155
A	RNA processing and modification	1	1
K	Transcription	113	115
L	Replication, recombination and repair	99	111
B	Chromatin structure and dynamics	3	3
D	Cell cycle control, cell division, chromosome partitioning	16	24
Y	Nuclear structure	0	0
V	Defense mechanisms	42	37
T	Signal transduction mechanisms	100	95
M	Cell wall/membrane/envelope biogenesis	106	88
N	Cell motility	12	31
Z	Cytoskeleton	0	0
W	Extracellular structures	0	0
U	Intracellular trafficking, secretion, and vesicular transport	24	22
O	Posttranslational modification, protein turnover, chaperones	92	97
C	Energy production and conversion	209	211
G	Carbohydrate transport and metabolism	90	85
E	Amino acid transport and metabolism	136	144
F	Nucleotide transport and metabolism	55	50
H	Coenzyme transport and metabolism	119	120
I	Lipid transport and metabolism	28	24
P	Inorganic ion transport and metabolism	106	103
Q	Secondary metabolites biosynthesis, transport and catabolism	11	14
R	General function prediction only	309	323
S	Function unknown	282	304
-	Not in COGs	893	932
