# Supplementary material for: Genome Sequence of a Mesophilic Hydrogenotrophic Methanogen Methanocella paludicola, the First Cultivated Representative of the Order Methanocellales
Source: PLoS One. 2011 Jul 29;6(7):e22898. doi: 10.1371/journal.pone.0022898 (PMC3146512; doi:10.1371/journal.pone.0022898)
Supplement: Table S2 — Sequence alignment of c/K subunits of V type ATPases. This table was made based on the data of Mulkidjanian et al. [24]. Active site residues are indicated in colored as follows: conserved ion-binding acidic (Glu/Asp) residue in red; other Na+ ligands are in right blue. The hydrophobic residue corresponding to Val63 of Ilyobacter tartaricus c subunit is colored orange. The conserved small (Pro, Gly, Ala, Ser) residue, corresponding to Pro28 of I. tartaricus c subunit is colored pink. Predicted cation specificity of the c/K subunit. Ions whose binding has been experimentally studied are shown in bold. (PDF) [file pone.0022898.s005.pdf]

Table S2. Sequence alignment of c/K subunits of V type ATPases.

Microorganism		Locus_tag	Residues	Sequence (aligned)*	Ion†
Methanocellales	*Methanocella paludicola*	MCP_0343	10-69	GAGLAVGLAGIGSGIAEKDIGAAAVGAIAEDRSFFGQGLIFTVIPETIVIFGLVIAILLM	H^+
		MCP_2286	9-68	GAAIAFGAGAISTGFAQARIGSAGAGALSERPELSGLIIILEAIPETLAILGFVVAAMIM	
	Uncultured methanogenic archaeon RC-I	RCIX2026	9-68	GVGLAVGLAGIGTGMSQGPIGAGVVGAVAEDRSFLGMGVFFIALPETIVIFGLVFAILLM	H^+
Methanosarcinales	*Methanosaeta thermophila*	Mthe_1613	16-75	GAGLATGLAGIGAGVGEQGIGAAVVGVVAEEPGFLGKGLFLMLLPETLIIFGLAVSLILM	H^+
	Methanosarcina barkeri	Mbar_A0378	20-79	GASIAIALTGIASAIAEKDIGTAAIGAMAENEGLFGKGLILTVIPETIVIFGLVVALLIN	**H^+**
		Mbar_A0390	20-79	GASIAIALTGIASAIAEKDIGTAAIGAMAENEGLFGKGLILTVIPETIVIFGLVVALLIN	
	Methanosarcina mazei	MM_0784	20-79	GAAIAIAVTGLASAIAEKDIGTAAIGAMAENEGLFGKGLILTVIPETIVIFGLVVALLIN	H^+
Methanococcales	*Methanocaldococcus jannaschii*	MJ_0221	11-70	GAGLAVGIAGLGSGIGAGITGASGAGVVAEDPNKFGTAIVFQALPQTQGLYGFLVAILIL	Na^+
		MJ_0221	83-141	AAGLAAGLAGL-SAIGQGIAASAGLGAVAEDNSIFGKAMVFSVLPETQAIYGLLIAILLL	
		MJ_0221	158-216	GAGFAVGFAGL-SGIGQGITAAGAIGATARDPDAMGKGLVLAVMPETFAIFGLLIAILIM	
Methanobacteriales	*Methanothermobacter thermautotrophicus*	MTH_959	14-73	GAGVAVGFAGLGSGLGQGIAAAESVGAVAENSDMFARGIIFSTLPETQAIYGFLIAILLL	**Na^+**
		MTH_959	95-154	GAGAAIGFAGLGSGMGQGITSASSVGAVVEDPDMFARGIIFSALSETQAIYGFLIAILLM	
	Ilyobacter tartaricus	2BL2_A	18-77	AMATATIFSGIGSAKGVGMTGEAAAALTTSQPEKFGQALILQLLPGTQGLYGFVIAFLIF	**Na^+**
		2BL2_A	94-153	GASLPIAFTGLFSGIAQGKVAAAGIQILAKKPEHATKGIIFAAMVETYAILGFVISFLLV	
