# Supplementary material for: Genome Sequence of a Mesophilic Hydrogenotrophic Methanogen Methanocella paludicola, the First Cultivated Representative of the Order Methanocellales
Source: PLoS One. 2011 Jul 29;6(7):e22898. doi: 10.1371/journal.pone.0022898 (PMC3146512; doi:10.1371/journal.pone.0022898)
Supplement: Table S3 — Carbon isotope fractionation of M. paludicola. Control indicates the value for the non- labeled cells. (PDF) [file pone.0022898.s006.pdf]

Table S3. Carbon isotope fractionation of *M. paludicola*.

	M. paludicola	
	Carbon content (mg)	δ^{13}C-PDB (‰)
Control	1.04	-32.4
5atm% $^{13}CH_3{}^{13}COONa$	0.901	1534.3
5atm% $NaH^{13}CO_3$	1.18	861.9
