# Supplementary material for: Genome Sequence of a Mesophilic Hydrogenotrophic Methanogen Methanocella paludicola, the First Cultivated Representative of the Order Methanocellales
Source: PLoS One. 2011 Jul 29;6(7):e22898. doi: 10.1371/journal.pone.0022898 (PMC3146512; doi:10.1371/journal.pone.0022898)
Supplement: Table S4 — Antioxidant enzymes among methanogens. The number indicates multiple gene copies. -, not present; n.d., not determined. Catalases indicated are E (katE, type I monofunctional clade II large subunit hemed catalase), A (katA, type I monofunctional clade III small subunit hemeb catalase), and G (katG, type II bifunctional hemeb catalase/peroxidase). Type of superoxide dismutases indicated are C (sodC, Cu-Zn-containing periplasmic enzyme) and B (sodB, Fe-containing cytoplasmic enzyme). (PDF) [file pone.0022898.s007.pdf]

Table S4. Antioxidant enzymes among methanogens.

Organism	Catalase (kat)	Superoxide dismutase (sod)	1-Fe superoxide reductase (sor)	2-Fe superoxide dismutase, desulfoferrodoxin (dfx)	Rubrerythrin (rbr)	peroxiredoxin (prx)	$F_{420}H_2$ oxidase (fprA)
Methanocellales							
M. paludicola SANAE	-	-	1×	-	2×	4×	1×
RC-I_{MRE50}	E	C	1×	1×	2×	5×	3×
Methanosarcinales							
M. mazei Go1	A+E	B	1×	-	2×	3×	1×
M. acetivorans C2A	G	B+C	1×	-	2×	3×	2×
M. barkeri str. Fusaro	A	B+C	-	-	2×	2×	2×
Methanomicrobiales							
M. hungatei JF-1	G	B	-	1×	2×	2×	2×
M. marisnigri JR1	E	B	-	-	1×	2×	2×
M. labreanum Z	A	-	-	1×	3×	1×	1×
M. boonei 6A8	G	2xB	-	1×	2×	1×	2×
M. palustris E1-9c	G	-	-	1×	2×	2×	2×
Methanobacteriales							
M. thermautotrophicus ΔH	-	B	-	1×	2×	1×	3×
M. stadtmanae DSM 3091	-	-	-	1×	1×	-	1×
Methanococcales							
M. jannaschii DSM 2661	-	-	1×	-	1×	1×	3×
M. maripaludis S2	-	-	1×	-	1×	1×	3×
Methanopyrales							
M. kandleri AV19	-	-	-	-	-	-	-
