# Supplementary material for: Genome Sequence of a Mesophilic Hydrogenotrophic Methanogen Methanocella paludicola, the First Cultivated Representative of the Order Methanocellales
Source: PLoS One. 2011 Jul 29;6(7):e22898. doi: 10.1371/journal.pone.0022898 (PMC3146512; doi:10.1371/journal.pone.0022898)
Supplement: Table S5 — The number of genes of histidine kinase and response regulator of M. paludicola and RC-IMRE50. (PDF) [file pone.0022898.s008.pdf]

Table S5. The number of genes of histidine kinase and response regulator of *M. paludicola* and RC-I_{MRE50}.

	M. paludicola	RC-I_{MRE50}
Histidine kinase	53	45
Response regulator	9	14
Hybrid of histidine kinase and response regulator	1	0
Domain architecture of histidine kinase		
HATPase_c	5	3
PAS-HATPase_c	13	14
PAS-PAS-HATPase_c	8	15
PAS-PAS-PAS-HATPase_c	9	1
PAS-PAS-PAS-PAS-HATPase_c	6	1
PAS-PAS-PAS-PAS-PAS-HATPase_c	1	0
PAS-PAS-GAF-HATPase_c	2	0
PAS-GAF-PAS-HATPase_c	2	0
GAF-HATPase_c	2	1
GAF-HisKA-HATPase_c	1	0
GAF-PAS-PAS-HATPase_c	1	0
Cache_1-HAMP-PAS-HATPase_c	2	0
HAMP-PAS-HATPase_c	0	2
HAMP-HATPase_c	1	8
Response_reg-PAS-HATPase_c	1	0
