# Supplementary material for: Genome Sequence of a Mesophilic Hydrogenotrophic Methanogen Methanocella paludicola, the First Cultivated Representative of the Order Methanocellales
Source: PLoS One. 2011 Jul 29;6(7):e22898. doi: 10.1371/journal.pone.0022898 (PMC3146512; doi:10.1371/journal.pone.0022898)
Supplement: Table S6 — Distribution of shared genes of M. paludicola and RC-IMRE50 with other methanogenic archaeal genomes. Asterisks show the value indicates the number and percentage of the genes of M. paludicola and RC-IMRE50 shared with each methanogen genome. (PDF) [file pone.0022898.s009.pdf]

Table S6. Distribution of shared genes of *M. paludicola* and RC-I_{MRE50} with other methanogenic archaeal genomes.

Order	Species	No. of CDS	*M. paludicola*		RC-I_{MRE50}	
			No. and percentage of shared genes*		No. and percentage of shared genes*	
Methanocellales	*M. paludicola* SANAE	3004	-	-	2059	66.7%
	RC-I_{MRE50}	3085	2100	69.9%	-	-
Methanosarcinales	*M. acetivorans* C2A	4540	1370	45.6%	1388	45.0%
	M. barkeri str. Fusaro	3606	1343	44.7%	1355	43.9%
	M. mazei Go1	3370	1365	45.4%	1374	44.5%
	M. burtonii DSM 6242	2273	1224	40.7%	1198	38.8%
	M. thermophila PT	1696	1121	37.3%	1078	34.9%
Methanomicrobiales	*M. marisnigri* JR1	2489	1282	42.7%	1254	40.6%
	M. palustris E1-9c	2655	1267	42.2%	1264	41.0%
	M. boonei 6A8	2450	1225	40.8%	1231	39.9%
	M. hungatei JF-1	3139	1230	40.9%	1228	39.8%
	M. labreanum Z	1739	1052	35.0%	1037	33.6%
Methanobacteriales	*M. thermautotrophicus* ΔH	1873	1046	34.8%	1010	32.7%
	M. smithii ATCC 35061	1793	962	32.0%	927	30.0%
	M. stadtmanae DSM 3091	1534	924	30.8%	890	28.8%
Methanococcales	*M. jannaschii* DSM 2661	1729	903	30.1%	901	29.2%
	M. maripaludis S2	1722	1038	34.6%	1019	33.0%
Methanopyrales	*M. kandleri* AV19	1687	785	26.1%	775	25.1%
